# The long noncoding RNA HOTAIR has tissue and cell type-dependent effects on HOX gene expression and phenotype of urothelial cancer cells

**DOI:** 10.1186/s12943-015-0371-8

**Published:** 2015-05-21

**Authors:** Judith Heubach, Juliana Monsior, René Deenen, Günter Niegisch, Tibor Szarvas, Christian Niedworok, Wolfgang Arthur Schulz, Michèle Janine Hoffmann

**Affiliations:** Department of Urology, Medical Faculty, Heinrich-Heine-University Duesseldorf, Moorenstr. 5, 40225, Duesseldorf, Germany; Biomedical and Medical Research Center, Heinrich-Heine-University Duesseldorf, Universitaetsstr. 1, 40225 Duesseldorf, Germany; Department of Urology, University of Duisburg- Essen, Hufelandstr. 55, 45147 Essen, Germany; Department of Urology, Semmelweis University, Ülloi ut 78/b, 1082 Budapest, Hungary

**Keywords:** HOTAIR, Long noncoding RNA, Bladder cancer, HOX gene

## Abstract

**Background:**

Urothelial carcinoma (UC) is the fifth most common cancer in the developed world. Delineation of differentiation subtypes in UC highlighted the importance of aberrant differentiation. Understanding underlying mechanisms may facilitate diagnosis and development of efficient therapy strategies. It is well accepted that epigenetic mechanisms are involved. Long noncoding RNAs (lncRNAs), a new class of epigenetic factors, are thought to mediate molecular differences between cell types to control cellular identity. The present study focuses on the lncRNA HOTAIR, originating from the HOXC locus. Its overexpression induces an aggressive phenotype in many cancers and aberrant expression of homeotic HOX transcription factors, especially HOXD10, that regulate differentiation and tissue homeostasis. The aim of the present study was to determine the functional role of HOTAIR in UC with regard to aggressive phenotype, regulation of aberrant differentiation and altered HOX gene expression.

**Methods:**

We determined RNA expression levels of HOTAIR and HOX genes in UC tissues and cell lines. Knockdown of HOTAIR and ectopic overexpression was performed to determine the effect on reported target genes in UC. Cell lines were stably transfected with HOTAIR to investigate changes in phenotype and HOX gene expression.

**Results:**

HOTAIR was overexpressed in approximately half of UC tissues and cell lines. Effects of HOTAIR overexpression differed between cell lines. Whereas VM-CUB1 cells acquired the expected phenotype with increased proliferation, clonogenicity, anchorage independent growth, migratory activity and epithelial-to-mesenchymal transition, 5637 cells grew more slowly displaying induction of senescence and related immune response genes. Other UC lines showed intermediate effects. Expression profiling revealed divergent effects on HOX genes, cell cycle regulators and differentiation according with the phenotypic differences between HOTAIR-overexpressing VM-CUB1 and 5637 cells.

**Conclusions:**

Our data indicate that HOTAIR overexpression may affect differentiation state and aggressiveness of UC cells, but in a cell-type dependent manner. Our functional studies and the comparison of our expression data sets with those from other cancer cell types, which revealed minimal overlaps, indicate that effects of HOTAIR are strongly tissue-dependent and can even differ within one cancer type. Thus, HOTAIR functions and target genes cannot simply be transferred from one cancer type to the other.

**Electronic supplementary material:**

The online version of this article (doi:10.1186/s12943-015-0371-8) contains supplementary material, which is available to authorized users.

## Background

Bladder cancer is the fifth most common cancer in the developed world and accounts for almost 25.000 new cases per year in Germany [1]. Urothelial carcinoma (UC) can be further subdivided into subtypes which are distinct in both clinical and molecular respects [2]. In particular, muscle-invasive tumors face a poor prognosis with only 50 – 60% survival at 5 years [3]. Tumor heterogeneity in UC is now thought to derive from differences in the respective cancer stem cell populations and in the extent of aberrant differentiation, as determined by specific profiles of surface markers and cytokeratins. Differentiation subtypes and their cell populations possess different tumorigenic potential [4]. These findings call for the delineation of the mechanisms regulating normal and aberrant differentiation in the urothelium in order to improve prognostic classification and to develop new strategies for targeting the tumor-initiating cell populations as a driving force of progression, metastasis and recurrence [5].

Homeotic HOX genes build an important network regulating differentiation patterns. They are located on four clusters on different chromosomes and are expressed in a precise spatio-temporal pattern. The paralogous posterior HOX groups 11 and 13 are active during development of the urogenital system [6,7]. Importantly, positional information and cellular identity are specified by combinatorial HOX gene expression rather than by any individual locus. Disruption of such patterns in neoplasia leads to aberrant differentiation [8]. In UC, regulation and function of HOX genes have hardly been investigated.

The transcriptional regulation of HOX genes in vertebrates is not fully understood, but epigenetic mechanisms like histone modifications and DNA methylation are established as being crucial. Polycomb (PcG) and Trithorax (TrX) group complexes are key factors in this regulation. Recent findings indicate that they may be directed to their target genes by specific long noncoding RNAs (lncRNA). These transcripts also affect many further cellular processes by various functions in transcriptional and post transcriptional gene regulation as well as in regulating nuclear architecture [9,10]. Due to their expression patterns, which are highly tissue-specific and differentiation-state dependent, they are thought to mediate the fine tuning of cellular identity by amplifying and consolidating molecular differences between cell types [11]. Intriguingly, several lncRNAs are encoded within HOX gene clusters [12], and are likewise expressed in a tissue-specific manner and along developmental axes. The best studied of these lncRNAs, HOTAIR, is expressed from the posterior region of the HOXC cluster and is implicated in the regulation of development and in cancer. To date it is reported to interact mainly with the EZH2, SUZ12 and LSD1 histone-modifiers thereby mediating repressive histone modifications at H3K27 and H3K4 *in trans*, e.g. at posterior HOXD genes [12]. The central genes of the HOXC cluster, especially *HOXC5* and *HOXC6*, are reported to become aberrantly activated in UC [13], but HOTAIR has not been comprehensively studied yet. Very recently, its expression has been reported to be increased in stage Ta/T1 bladder cancers and to be correlated with recurrence [14]. HOTAIR is overexpressed in various cancers, e.g. of the breast, the lung, esophagus, pancreas and the gastrointestinal system and is usually associated with an aggressive phenotype [15-19]. However, this rather uniform effect is somewhat surprising given the postulated tissue-specific expression of lncRNAs and especially of their HOX target genes.

We therefore wondered whether the findings in other cancers on expression and function of HOTAIR could be extended to UC, in particular with regard to the regulation of aberrant differentiation patterns and HOX gene expression. We discovered that HOTAIR was overexpressed in approximately half of the investigated UC tissues and cell lines. We report that effects of ectopic HOTAIR overexpression on phenotype, differentiation and target genes differed between cell lines. Subsequent to modulation of HOTAIR expression by knockdown or ectopic overexpression, we did not observe the same effects on HOTAIR target genes among the posterior HOXD genes as previously reported for other cancer types. Further, comparisons of our gene expression microarray data, obtained from cell lines with ectopic HOTAIR overexpression, with those from similar experiments with cell lines from other cancer types revealed minimal overlap, suggesting that effects of HOTAIR are strongly tissue-dependent and cannot simply be extrapolated from one cancer type to another.

## Results

### HOX C and HOX D genes are aberrantly expressed in urothelial carcinoma

First, we determined endogenous HOTAIR expression levels and in parallel the expression of HOX genes by quantitative real time PCR. We chose posterior HOXC genes *HOXC11-13* located in close proximity to the HOTAIR transcript and posterior HOXD genes *HOXD10-13*, reported to be regulated by HOTAIR in normal fibroblasts and breast cancer mediating aggressive phenotype [12,15]. Furthermore, we assessed overexpression of the *HOXC6* gene from the center of the HOXC locus to ascertain that our sample set was representative [13]. Expression of these nine genes was determined in a set of 19 UC tissues compared to 10 normal bladder tissues (designated Set 1) and in UC cell lines compared to cultured normal uroepithelial cells (UEC). The mammary cancer cell line MCF7 was included for comparison with published data for breast cancers [15].

**Figure 1:**
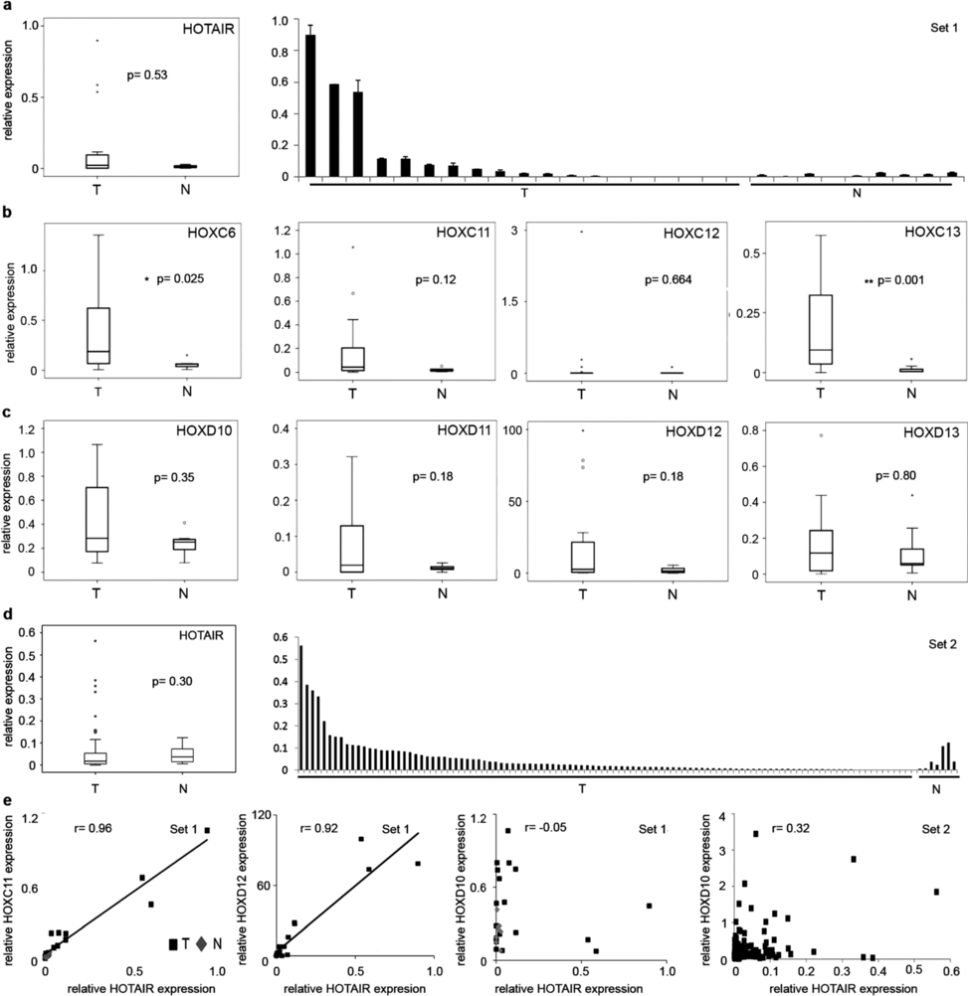
Expression of HOTAIR, HOXC and HOXD genes in benign and cancerous urothelial tissues. **(a)** Boxplot graph illustrating expression level of HOTAIR in UC tissue sample set 1 (T, n = 19) as compared to normal bladder tissues (N, n = 10; p = 0.53). Expression was measured by quantitative real-time PCR and normalized to *TBP.* The range of HOTAIR expression levels among UC tissues is illustrated in the second graph with the highest HOTAIR expression found in three pT3 high grade tumors. **(b, c)** Boxplot graphs for indicated genes in tissues of set 1 (p-values as indicated; *p ≤ 0.05, **p ≤ 0.001). **(d)** HOTAIR expression levels for a second set of UC tissue samples (Set 2, n: T = 108, N = 7) shown as boxplot diagram (p = 0.3) and as a range of expression levels differing among patients. **(e)** Correlation between expression of HOTAIR and *HOXC11*, *HOXD10* and *HOXD12* is plotted, where “r” denotes the Pearson correlation coefficient. Black squares denote tumor samples, grey rhombs denote benign tissues.

We found HOTAIR expression to be increased in about half of the UC tissues (9/19; Figure 1a) and particularly highly overexpressed in three progressive muscle-invasive bladder carcinomas (all pT3 high grade). However, we found no further association between increased HOTAIR expression and tumor stage due to the small cohort size of this sample set. Significant reactivation of the *HOXC6* gene in UC validated our sample set as representative (Figure 1b, p = 0.025). For the posterior HOXC genes we observed a significant reactivation of *HOXC11* and *HOXC13* in tumor tissues (Figure 1b, *HOXC13* p = 0.001). *HOXC11* expression was well correlated with HOTAIR expression in tumor tissues (r Pearson = 0.96, Figure 1e). In contrast, *HOXC12* was not expressed, indicating that the function of the boundary located between *HOXC11* and *HOXC12* was maintained. *HOXD10* and *HOXD11* were expressed at detectable levels in normal bladder tissues (Figure 1c), and more strongly in tumor tissues, with no evidence for the expected inverse correlation between HOTAIR and *HOXD10* expression (Figure 1e) [12,15]. Furthermore, we found reactivation of *HOXD12* and *HOXD13* expression in selected cancer samples (Figure 1c) and, surprisingly, a strong positive correlation between *HOTAIR* and *HOXD12,* particularly in overexpressing UC tissues (r Pearson = 0.92, Figure 1e). Thus, we did not observe any inverse correlation between HOTAIR and *HOXD10* expression, neither in our own sample set (Set 1, r Pearson = −0.05) nor in a second validation set (Set 2, r Pearson = 0.32; Figure 1e).

**Figure 2:**
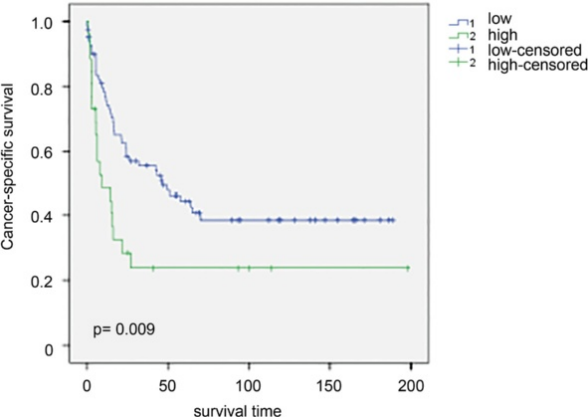
Kaplan-Meier-analysis for HOTAIR expression and cancer-specific survival. Kaplan-Meier-analysis revealed a significant advantage with regard to cancer-specific survival (p = 0.009) for patients with low HOTAIR expression (including 75% of patients with low or average HOTAIR expression levels) when compared with the 25% of patients with highest HOTAIR expression levels. Survival time is given in months.

This second set comprised a larger number of tissue samples (n = 108) and revealed a similar range of HOTAIR expression among the tumors (Figure 1d) as observed for Set 1 (Figure 1a). Despite the larger cohort size, we did not observe a significant correlation between overall HOTAIR expression and any clinicopathological parameter (Table 1). As we observed that only about 20% of patients displayed significantly higher levels of HOTAIR (>2-fold than the median of normal tissues, Figure 1d), we grouped the patients into two groups according to their HOTAIR expression level for further statistical analysis (group 1 = 25% of patients with higher HOTAIR expression, group 2 = 75% of patients with average or low HOTAIR expression). Between these groups, Kaplan-Meier-analysis displayed a significant longer cancer-specific survival for patients with low HOTAIR expression (p = 0.009, Figure 2). Moreover, this stratification revealed a significant correlation between top 25% HOTAIR expression levels and tumor stage, grade or lymph node metastasis in univariate and multivariate analyses (Table 2).

**Table 1:** **HOTAIR expression in relation to clinicopathological characteristics of UC Set 2**

**Variables**			
		**HOTAIR expression**	
	**n**	**Median (range)**	**P**
Age			
≤65	54	1.95 (0.00 - 56.20)	0.822
>65	54	1.70 (0.00 - 35.90)	
Gender			
Male	79	1.70 (0.00 - 56.20)	0.501
Female	29	1.80 (0.10 - 14.90)	
Stage			
Ta	14	2.45 (0.10 - 15.70)	
T1	13	2.60 (0.10 - 56.20)	
T2	17	1.70 (0.00 - 15.00)	
T3	44	1.50 (0.10 - 38.50)	
T4	20	1.50 (0.00 - 33.20)	
Non-inv.	27	2.50 (0.10 - 56.20)	0.614
Invasive	81	1.60 (0.00 - 38.50)	
Grade			
G1	7	2.50 (0.10 - 15.70)	
G2	33	2.60 (0.00 - 35.90)	
G3	68	1.35 (0.00 - 56.20)	
Low-grade (G 1–2)	40	2.55 (0.00 - 35.90)	0.256
High-grade (G 3)	68	1.35 (0.00 - 56.20)	
Lyph node			
N0/Nx/M0/Mx	80	1.60 (0.00 - 35.90)	0.766
N + / M+	28	1.20 (0.10 - 38.50)	
Smoking			
yes	61	2.40 (0.00 - 56.20)	0.832
no	29	2.10 (0.10 - 35.90)	
unknown	18

**Table 2:** Univariate and multivariate analysis for HOTAIR expression and DSS of patients from UC Set 2 (n = 108)

***Variables***	**Disease-specific survival**		
	**HR**	**95% CI**	**P**
*Univariate analysis*			
Age (≤60 vs > 60 years)	0.923	0.562 - 1.516	0.753
Gender (male vs female)	0.697	0.407 - 1.195	0.190
Tumor stage (Ta-T1 vs T2-T4)	5,401	2.321 - 12.568	**<0.001**
Grade (G1- G2 vs G3)	4,237	2.203 - 8.150	**<0.001**
Lymph node metastasis (yes vs no)	4,884	2.832 - 8.422	**<0.001**
HOTAIR expression > 75% (high vs low)	2,033	1.180 - 3.501	**0.011**
*Multivariate analysis*			
Stage (T2-T4)	2,654	1.021 - 6.856	**0.045**
Grade (G3)	2,327	1.115 - 4.854	**0.024**
Lymph node metastasis (N+)	2,951	1.661 - 5.242	**<0.001**
HOTAIR expression (>75%)	2,200	1.232 - 3.928	**0.008**

*Abbreviations*: *DSS-* disease-specific survival; *HR* hazard ratio, *CI* confidence interval.

Patients were dichotomised according to their HOTAIR expression level (group 1 = 25% of patients with highest HOTAIR expression, group 2 = 75% of patients with average or low HOTAIR expression).

**Figure 3:**
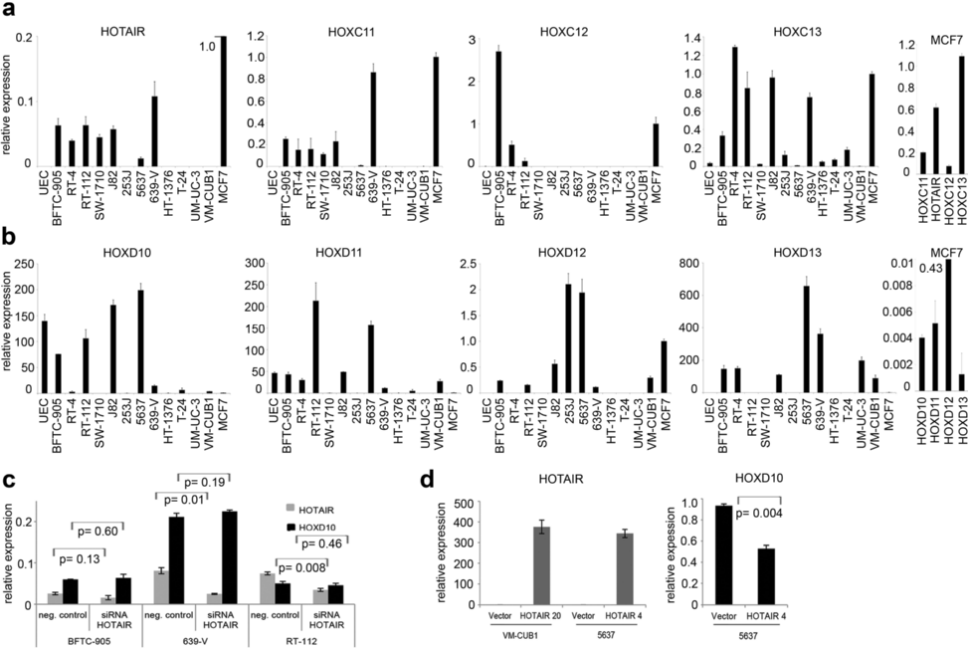
HOX gene expression in UC cells and cell type dependent effects of HOTAIR modulation. **(a, b)** mRNA expression of HOTAIR, posterior HOXC and HOXD genes was determined in UC cell lines and six different cultures of normal proliferating uroepithelial cell cultures (UEC; average expression level illustrated) by qRT-PCR and normalized to *TBP*. Expression levels are shown relative to the mammary cancer cell line MCF7, which was used as a control and set as 1. Expression values exceeding the scale are given as numerical values. To show the activity of HOX loci in the MCF7 cell line, expression levels of HOXC and HOXD genes are also illustrated separately for this cell line. Relative expression of HOTAIR (grey column) and *HOXD10* (black column) was determined by qRT-PCR after siRNA-mediated knockdown of HOTAIR in three different bladder cancer cell lines **(c)** or following overexpression of HOTAIR in stable clones from VM-CUB1 and 5637 cell lines **(d)**. Expression of *HOXD10* in VM-CUB1 remained at background levels despite HOTAIR overexpression and is not illustrated.

In UC cell lines, HOTAIR expression was also increased in about half of the cell lines (6/11) compared to the average expression of six independent cultures of normal uroepithelial cells (UEC, Figure 3a). Of note, HOTAIR expression in UC cell lines was not quite as high as in the mammary cancer cell line MCF7, which was used as a control for very high expression. MCF7 generally displayed higher expression of posterior HOXC genes compared to bladder cancer cell lines, including *HOXC12* which is silenced in most UC cells (Figure 3a).

The expression pattern of posterior HOXD genes was heterogeneous in UC cell lines. *HOXD10* was completely silenced in the MCF7 control cell line, but was highly expressed in several UC cell lines (Figure 3b), even though they overexpressed HOTAIR (Figure 3a). *HOXD10* was also strongly expressed in all six cultures of UEC (Figure 3b), suggesting that this locus remains important for tissue homeostasis in the adult urogenital system. In other UC cell lines *HOXD10* remained undetectable, independent of whether HOTAIR was present, suggesting that posterior HOXD genes may not be HOTAIR target genes in UC. Moreover, other posterior HOXD genes were highly expressed in some UC cell lines as compared to the MCF7 cell line with its high HOTAIR expression and almost complete silencing of posterior HOXD genes, except for *HOXD12* (Figure 3b).

### The regulatory function of HOTAIR in HOX gene regulation is cell-type dependent

To further elucidate the function of HOTAIR in the regulation of HOX gene expression in UC cells, we performed siRNA-mediated knockdown of HOTAIR expression and determined the effect on its presumable target gene *HOXD10* (Figure 3c). Although HOTAIR expression was significantly diminished to about 50% by two previously published siRNAs in two different cell lines (639-V p = 0.01, RT-112 p = 0.008), *HOXD10* expression was not induced (Figure 3c). Analogous results were obtained using LNA-antisense oligonucleotides (data not shown; see Additional file 1 for data on efficiency of LNA-based knockdown).

As some regulatory effects of HOTAIR in breast cancer cells occurred only after stable transfection and long-term serial passaging [15], we generated clones of 5637 and VM-CUB1 stably overexpressing HOTAIR as well as vector controls. Both UC lines are moderately differentiated and share an epithelial phenotype and low HOTAIR expression, but posterior HOXD genes are more strongly expressed in 5637 than in VM-CUB1.

From clones with various levels of HOTAIR overexpression, we used mostly those with the highest levels to mimic HOTAIR expression in breast cancer (Figure 3d). Clones overexpressing at intermediate level were later used to further validate expression profiling results and to investigate dose dependency. *HOXD10* expression remained low in HOTAIR-transfected VM-CUB1 cells, but was diminished in 5637 cells overexpressing HOTAIR (5637_HOTAIR 4; Figure 3d, p = 0.004).

### The induction of an aggressive phenotype by HOTAIR overexpression is cell-type dependent

**Figure 4:**
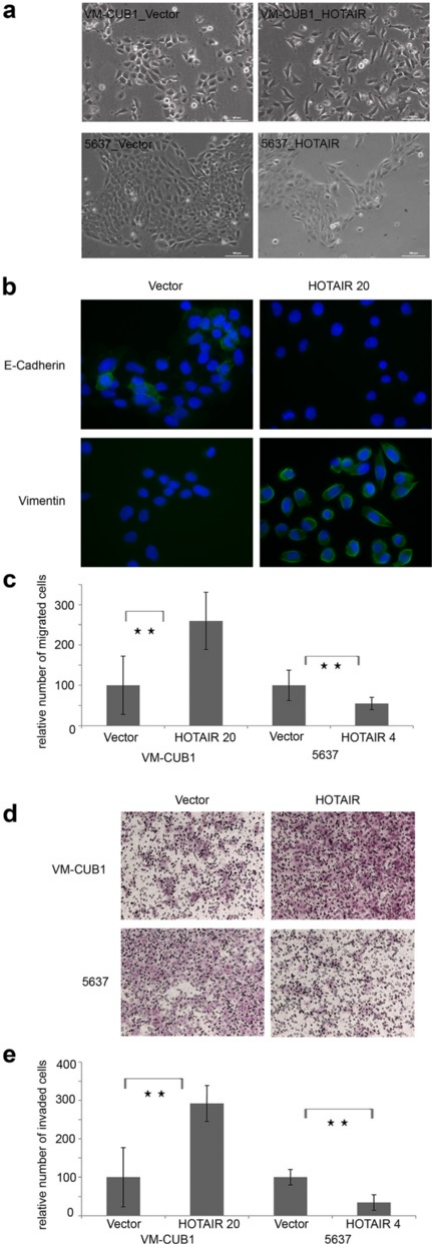
Cell type dependent induction of an aggressive phenotype in VM-CUB1 cells. **(a)** Phenotypical changes of stably transfected VM-CUB1 and 5637 clones as seen in light microscopy (10x magnification). **(b)** Immunofluorescence stainings for E-Cadherin (top, green) and Vimentin (bottom, green) to verify EMT induction in VM-CUB1-HOTAIR 20 cells (40 x magnification). Transwell assays were performed to determine changes in migration **(c)** and invasion **(d, e)** capacity (**p ≤ 0.001). Data results from one assay with 4 transwells per cell line and 5 visual fields, which were randomly chosen for counting the number of migrated or invaded cells. Results are representative for assays repeated in triplicate. Invaded cells were visualized by H&E staining (**d**, 10 x magnification).

We analyzed HOTAIR clones of the two cell lines for the aggressive phenotype described in other tumor types [15-19]. Differences between the HOTAIR clones of the two cell lines became already evident during cell culturing. While 5637_HOTAIR clones retained their morphological phenotype (Figure 4a, bottom) but grew more slowly than control cells, VM-CUB1_HOTAIR clones grew faster and assumed a more mesenchymal phenotype with numerous filopodia and lamellipodia, indicative of EMT (Figure 4a). Indeed, whereas E-Cadherin staining was diminished compared to control cells, VM-CUB1_HOTAIR cells gained Vimentin expression (Figure 4b). Likewise, VM-CUB1_HOTAIR cells, but not 5637_HOTAIR cells displayed significantly increased migration (Figure 4c, p ≤ 0.001) and invasion capacity in transwell assays (Figure 4d, e, p ≤ 0.001).

**Figure 5:**
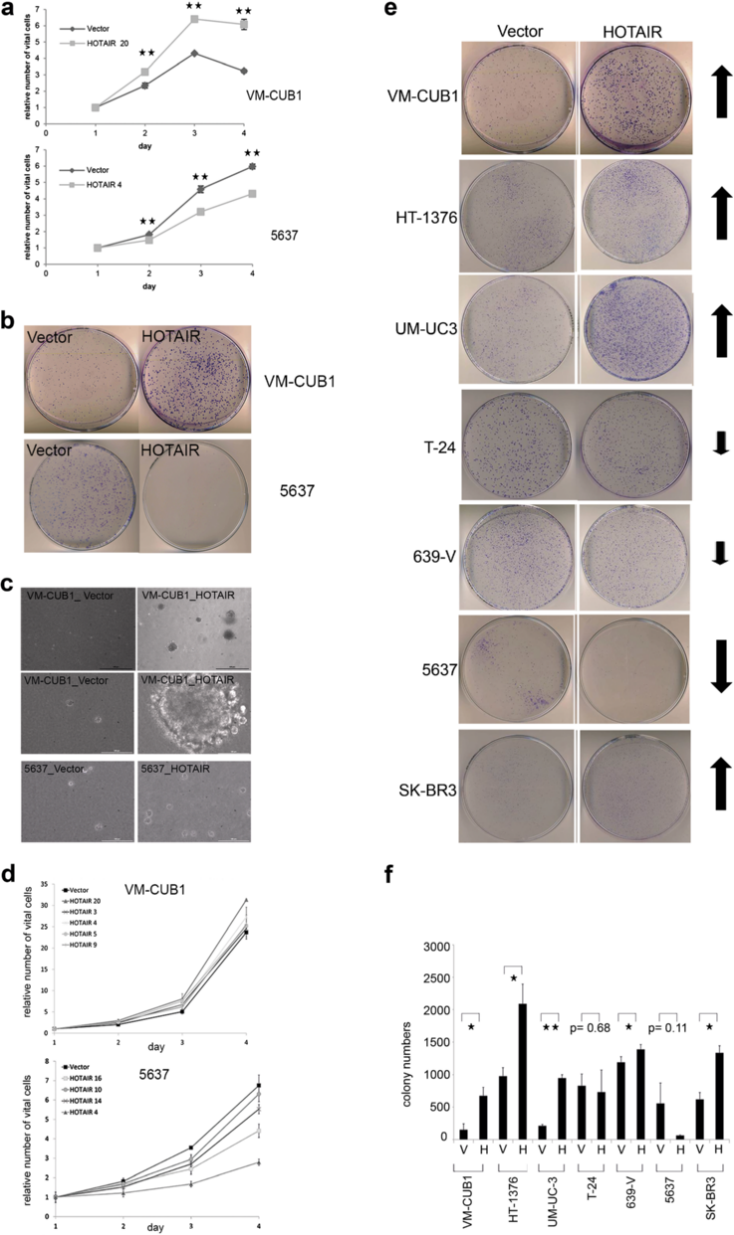
Cell type dependent effects of HOTAIR overexpression on proliferation and clonogenicity. **(a)** The relative number of vital cells was monitored in quadruplicates of stably HOTAIR-transfected VM-CUB1 and 5637 cell clones by MTT assay over 4 days. Results shown for one assay are representative for three independent assays (**p ≤ 0.001). **(b)** Effects of HOTAIR transfection on clonogenicity in VM-CUB1 or 5637 cells. **(c)** Representative picture from increased anchorage-independent growth of VM-CUB1_HOTAIR 20 cells in triplicate soft agar assays (4 x (top) and 20 x (center) magnification); 5637 cells poorly formed colonies independent of HOTAIR overexpression (20x, bottom). **(d)** MTT assays for additional stably transfected HOTAIR clones of VM-CUB1 and 5637, with analogous results to those shown in **(a)**. Number of vital cells was determined in quadruplicate at every time point for every clone. For p-values see Additional file 2: Table S1. **(e, f)** Cell-type dependent effects of HOTAIR on clonogenicity in further UC cell lines (*p ≤ 0.05, ** ≤ 0.001).

Similarly, whereas VM-CUB1_HOTAIR cells displayed significantly increased proliferation compared to vector-transfected controls, 5637_HOTAIR cells grew more slowly than control cells (Figure 5a, p ≤ 0.001). Analogous results were obtained in a clonogenicity assay (Figure 5b). In an anchorage-independent growth assay, neither HOTAIR-overexpressing nor 5637 control cells grew in soft agar, whereas VM-CUB1_HOTAIR cells generated larger colonies than the parental cells (Figure 5c). Four additional stably transfected VM-CUB1_HOTAIR clones also displayed increased proliferation, three additional stably transfected 5637_HOTAIR clones showed reduced proliferation (Figure 5d, for p-values see Additional file 2: Table S1). Cell type-dependent effects of HOTAIR were also obtained with further UC cell lines. Proliferation and clonogenicity increased in some HOTAIR transfected cell lines, but did not change or were diminished in others (Figure 5e, f). The mammary cancer cell line SK-BR3 served as a control in these experiments, yielding the expected increases.

### HOTAIR target genes differ between cell types and tissues explaining divergent effects

**Figure 6:**
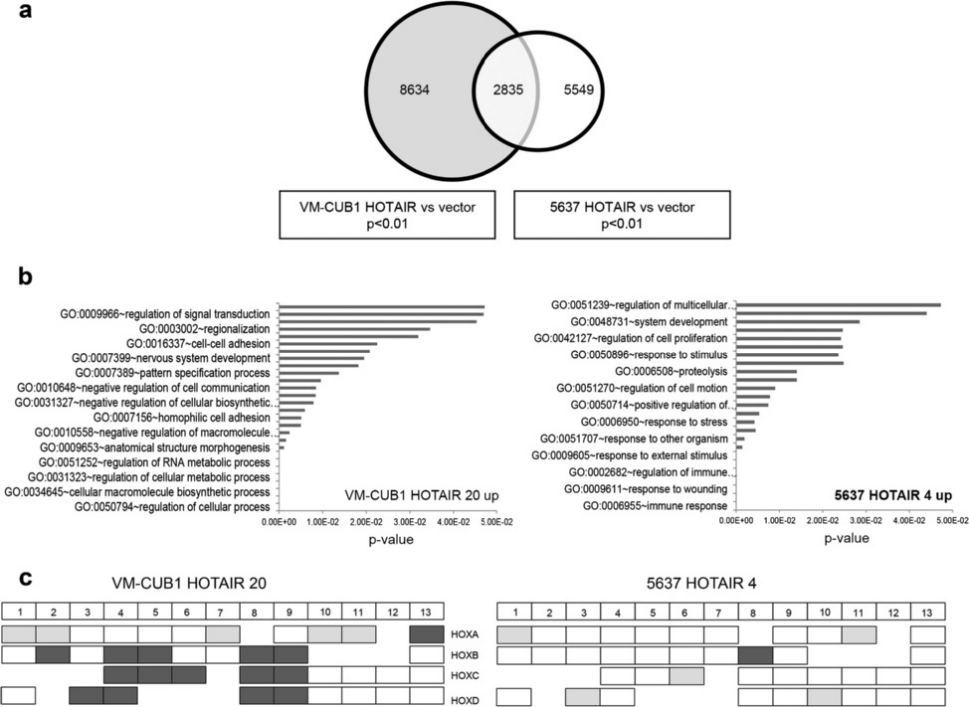
Results of RNA expression profiling reveal cell type dependent target genes of HOTAIR. **(a)** Venn diagram for differentially expressed genes in VM-CUB1_HOTAIR 20 and 5637_HOTAIR 4 cells when compared to the respective vector controls. Three independent RNA preparations of each clone were hybridized to Affymetrix PrimeView 3′IVT arrays. Differentially expressed genes with a significance of p < 0.01 (Benjamini-Hochberg adjusted) were selected. **(b)** Genes with at least two-fold overexpression in VM-CUB1 and 5637 (p < 0.01) were subjected to GO analysis using DAVID analysis software. GO biological process terms significantly enriched at p < 0.05 after adjustment were considered; the list was edited for multiple similar terms. **(c)** Changes in HOX gene expression pattern in stably transfected VM-CUB1 and 5637 cells (p < 0.01) displayed as a heatmap. Downregulated genes are indicated by light grey boxes, upregulated genes by dark grey boxes. HOX genes not significantly changed are shown in white.

Expression profiling analysis was performed to identify the genes differentially affected by HOTAIR in stably transfected VM-CUB1 and 5637 cell clones. At a cut-off p-value < 0.01 (Benjamini-Hochberg adjusted) we identified 8634 differentially expressed genes in VM-CUB1 cells and 5549 genes in 5637 cells compared to the respective vector controls (Figure 6a). Interestingly, about half of the genes were downregulated or upregulated in either cell line (VM-CUB1: 4205 genes downregulated, 4429 genes upregulated; 5637: 2783 genes downregulated, 2766 genes upregulated). Of these, 2835 genes were common to both cell lines, but only 784 of these were downregulated, indicating that also transcription activating chromatin modifications may be mediated by HOTAIR. A Gene ontology database (GO) search using DAVID [20] revealed involvement of differentially expressed genes in RNA processing/splicing, cell division/chromosome partitioning and intracellular transport. The 838 genes concomitantly induced in both cell lines were significantly assigned to processes like cellular protein localization, locomotion, regulation of cell death and JAK-STAT signaling. The remaining 1213 overlapping genes were regulated in opposite directions and significantly associated with GO groups like regulation of proliferation, regulation of apoptosis and metabolic processes concurring with the observed differing phenotypes. Interestingly, two HOX genes, *HOXD3* and *HOXC6*, were as well regulated in opposite directions.

Considering only at least two-fold expression changes reduced the number of differentially expressed genes to 3728 in VM-CUB1 cells and to 1195 in 5637 cells. The GO analysis results for these genes fitted even better to the divergent phenotypes with regard to proliferation, EMT-induction, migration and invasion capacity (Additional file 2: Table S2). Among the upregulated genes, significant terms concerned regulation of transcription and RNA metabolism, signal transduction, cell adhesion and morphogenesis (Figure 6b). Unexpectedly, the most significant group of genes upregulated in 5637 was involved in immune and inflammatory responses.

**Figure 7:**
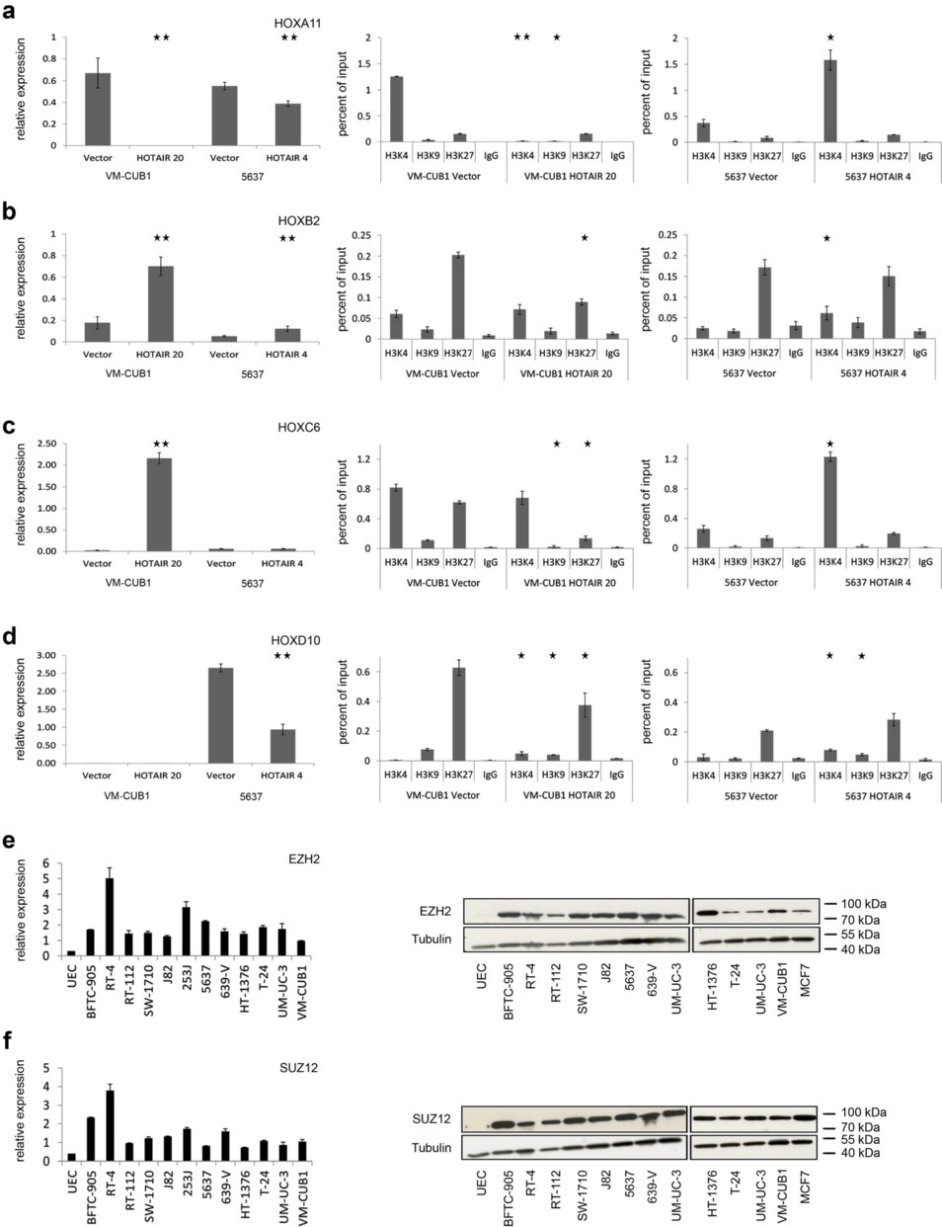
Mechanistic investigation of aberrantly expressed HOX genes by ChIP and qRT-PCR. **(a)** Differential expression of *HOXA11* in VM-CUB1 and 5637 HOTAIR clones was verified by qRT-PCR relative to TBP (left graph). ChIP with subsequent qPCR was used to determine changes in H3K4, H3K9 and H3K27 trimethylation, each as compared to IgG control for VM-CUB1 cells (middle) and 5637 cells (right). Analogous analyses for *HOXB2* **(b)**, *HOXC6* **(c)** and *HOXD10* **(d)**. Overexpression of polycomb group proteins EZH2 (**e**, 85 kDa) and SUZ12 (**f**, 83 kDa) on the RNA (left) and protein level (right) in UC cells. Tubulin protein (50 kDa) was detected as a loading control.

Downregulated genes in VM-CUB1_HOTAIR cells encoded cytokeratins (KRT5, 6A/B, 13, 14, 19, 80), *E-Cadherin* and negative cell cycle regulators. In contrast, EMT-inducing factors like *ZEB1*, and the EMT marker *Vimentin*, but also the hair keratins *KRT33B* and *34* were upregulated (Additional file 2: Table S3). In 5637_HOTAIR cells, conversely, EMT-inducing factors were downregulated, whereas CDK inhibitors and apoptosis-related genes were upregulated. Altered expression of interesting candidates was verified by quantitative real time RT-PCR (Additional file 2: Table S3, Figure 7). Furthermore, we analyzed clones with intermediate level ectopic HOTAIR overexpression for dose-dependency of the effects, which could only be detected for single candidates (Additional file 2: Table S3).

In particular, the effects of HOTAIR on HOX gene expression differed between both cell lines. While only a few HOX genes were affected in 5637 cells (n = 6, at p <0.01 Figure 6c), a large number of HOX genes was deregulated in VM-CUB1 cells. Five HOX genes were downregulated (light grey boxes) and 15 were upregulated (dark grey boxes); in accordance with the qRT-PCR results *HOXD10* was unaffected. Although *HOXD9* was previously reported to be repressed by HOTAIR in fibroblasts [12], it was 4.3-fold induced in VM-CUB1 cells. Similarly, all central HOXC genes were reactivated by HOTAIR in VM-CUB1, but posterior HOXC genes were not affected. In contrast, in 5637 cells, only *HOXB8* was induced and five HOX genes were downregulated, including *HOXD10. HOXA1* and *HOXA11* were repressed and *HOXB8* was induced in both cell lines. *HOXC6* and *HOXD3* were differently affected between the two cell lines (Figure 6c).

To elucidate whether differential expression of HOX genes was mediated by changes in histone H3 methylation, we performed quantitative chromatin immunoprecipitation (ChIP) for the *HOX* loci *A11*, *B2*, *C6* and *D10. HOXA11* was significantly downregulated by HOTAIR in VM-CUB1 cells, but only slightly diminished in 5637 cells. Downregulation was confirmed by qRT-PCR (Figure 7a), which also revealed dose dependency (Additional file 2: Table S3). Downregulation was associated with decreased H3K4 trimethylation in VM-CUB1 cells. *HOXB2* was upregulated in both cell lines, which was associated with diminished H3K27 trimethylation and slightly increased H3K4 methylation (Figure 7b). *HOXC6* was strongly induced in VM-CUB1 cells and marginally downregulated in 5637 cells. Its induction corresponded to diminished trimethylation of H3K27 and H3K9 (Figure 7c). The decreased expression of *HOXD10* in 5637 cells was accompanied by slight increases in H3K27 and H3K9 methylation (Figure 7d). As a potential cause of the differences between the cell lines, we furthermore determined the expression of two polycomb group proteins that mediate repressive histone modifications and interact with HOTAIR, namely EZH2 and SUZ12. We found broad overexpression of both factors at the mRNA and protein level in all investigated UC cell lines compared to UEC (Figure 7e,f), but no significant differences between VM-CUB1 and 5637.

**Figure 8:**
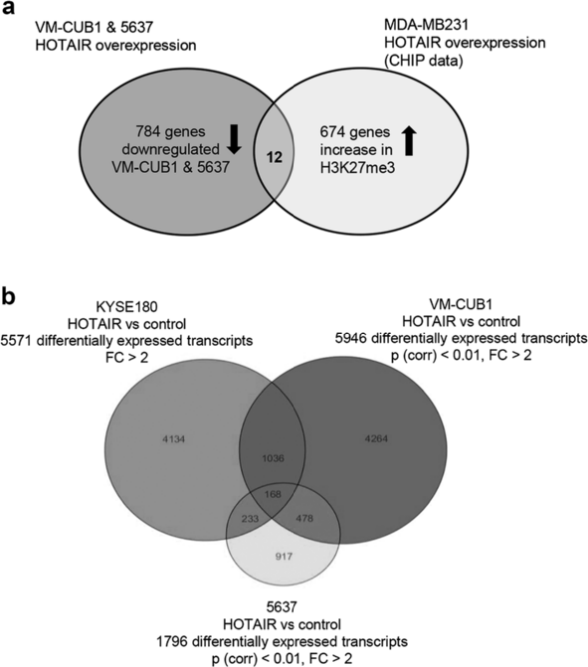
HOTAIR target genes are highly tissue-specific. **(a)** Publicly available ChIP data published by Gupta et al. [15] was used to select 674 genes gaining increased H3K27 trimethylation at promoters following ectopic expression of HOTAIR in MDA-MB-231 breast cancer cells. These were matched with 784 genes that were downregulated in both UC cell lines after stable ectopic expression of HOTAIR (p < 0.01). **(b)** Venn diagram for differentially expressed HOTAIR target genes in the esophageal cancer cell line KYSE180 [21] and the two UC cell lines VM-CUB1 and 5637 (p < 0.01) illustrating the minimal overlap of HOTAIR regulated genes between the two cancer types.

To further follow the indications that HOTAIR action might be cell type-dependent, we compared our results to those of others, especially to those for HOTAIR-overexpressing MDA-MB231 breast cancer cells [15]. Due to different study design and microarray platforms, we used the published ChIP rather than expression data, as in the comparison between PANC1 and MDA-MB-231 cells in ref. 18. To this end, we compared the 674 genes displaying an increase of the repressive histone modification H3K27me3 after HOTAIR overexpression in MDA-MB231 cells with the 784 genes downregulated in both 5637 and VM-CUB1 cells by HOTAIR expression (Figure 8a). This comparison revealed only 12 overlapping genes (*API5*, *CHD1L*, *COG2*, *COX6C*, *CRISPLD1*, *HEATR1*, *KIAA1324L*, *METRNL*, *PCTP*, *PLAGL1*, *RASAL2*, *ZNF287*), which therefore constitute candidates for tissue-independent HOTAIR target genes. Notably, regulation of all other genes by HOTAIR appears to be tissue-dependent.

We extended this analysis to reported HOTAIR target genes in pancreatic and esophageal cancer cells (Table 3) [18,21]. Again, this comparison revealed highly tissue-dependent effects of HOTAIR in the four tumor types with many genes being regulated in opposite directions. Interestingly, some immune response genes upregulated in HOTAIR transfected 5637 cells were also induced by HOTAIR in the pancreatic cancer cell line MiaPaC2 and the esophageal cancer cell line KYSE180. Importantly, detailed inspection revealed many genes that were differentially regulated between the MiaPaC2 and Panc28 pancreatic cancer cell lines, indicative of cell type-dependent effects within one cancer type like in the UC cell lines (Table 3) [18]. Finally, a comparison of gene expression changes in KYSE180 [21] with our own expression data revealed a minimal overlap of 168 genes in the intersection of all three cell lines (Figure 8b).

**Table 3:** **Expression of selected genes affected by ectopic HOTAIR overexpression according to Gupta et al.** [15]**, Kim et al.** [18] **and Li et al.** [21] **in comparison with microarray data from transfected bladder cancer cell lines**

	**Gupta et al. mamma**	**Kim et al. pancreas**	**Kim et al. pancreas**	**Li et al. esophagus**	**Heubach et al. bladder**	**Heubach et al. bladder**
	**MDA-MB-231**	**MiaPaCa2**	**Panc28**	**KYSE180**	**VM-CUB1**	**5637**
JAM2	down	down	up	down	--	--
PCDH10	down	down	up	down	--	--
PCDHB5	down	down	up	down	down	--
ABL2	up	down	up	n.c.	up	down
SNAIL1	up	up	up (n.s.)	down	down	down
LAMB3	up	down	n.c.	down	down	--
LAMC2	up	down	down (n.s.)	n.c.	down	down
GDF15	--	down	n.s.	up	up	up
IL29	--	down	up	n.c.	--	--
IL28A	--	down	n.s.	n.c.	--	--
IL28B	--	down	up	n.c.	--	--
IFTM1	--	up	n.s.	--	--	--
OAS1	--	up	n.s.	up	--	up
MX1	--	down	n.s.	up	down	up

*Abbreviations*: *n*.*s*. not significant, −- not detected.

## Discussion

The lncRNA HOTAIR is reported to be overexpressed in many different cancers and to contribute to an aggressive phenotype. In particular, HOTAIR is thought to regulate genes from the distal HOXD region *in trans*, especially *HOXD10*, which may mediate some of its effects. In this study we therefore investigated to which extent altered expression of HOTAIR contributes to altered HOX gene expression patterns and an aggressive phenotype in urothelial carcinoma. HOTAIR expression was increased in about half of the UC tissue samples from two independent cohorts and in UC cell lines, most pronounced in some high stage tumors. In the larger patient cohort we observed significant associations between the highest HOTAIR expression and cancer-specific survival as well as worse clinicopathological parameters. We did not observe the recently reported widespread increase of HOTAIR expression in pTa/pT1 bladder cancer tissues [14], but our cohort is derived from cystectomies and contains few cancers of those stages. Our findings rather fit well with the observation in breast cancer, where strongly increased HOTAIR expression was more prominent at higher stages and even more in metastases [15]. We also confirmed the reported overexpression of HOTAIR in breast cancer cell lines like MCF7, but nevertheless the effects of HOTAIR overexpression on HOX gene expression and on cellular properties were quite different between the two tumor entities and even non-uniform among individual UC cell lines.

As the expression pattern of HOX genes is specific to each tissue it would in fact be remarkable, if HOTAIR overexpression elicited the same response in each cancer type. For instance, in the posterior HOXC cluster, *HOXC11*-*C13* were more strongly expressed in MCF7 cells than in UC cell lines. These loci, with the exception of *HOXC12,* were upregulated together with HOTAIR in UC tissues and cell lines. However, a large number of HOX genes, especially central HOXC genes, were differentially expressed in stably transfected VM-CUB1_HOTAIR cells, but posterior HOXC loci *C10*-*C13* were not further upregulated. This finding suggests HOTAIR overexpression in UC represents a consequence of posterior HOXC reactivation, but is not the cause of the reactivation of posterior HOXC genes. Instead, HOTAIR overexpression might contribute to the upregulation of central HOXC genes, which is reportedly common in UC [13], as confirmed by our results. Ectopic HOTAIR increased expression of *HOXC4*-*9* in VM-CUB1 cells, although *HOXC6* was regulated by HOTAIR in the opposite direction in 5637 cells.

In contrast, posterior HOXD genes, especially *HOXD10*, were expressed in normal bladder tissue and uroepithelial cells, suggesting that they are involved in normal urothelial homeostasis. Nevertheless, these genes were aberrantly high expressed in several UC tissues and cell lines, including the 5637 cell line. In fibroblasts HOTAIR represses *HOXD8*-*D11* by recruiting PRC2 [12] and an inverse relationship between HOTAIR and *HOXD10* was reported in breast cancer [15]. These relations were observed neither in UC tissues nor cell lines. Instead, we discovered a positive correlation between HOTAIR and *HOXD12* in UC tissues. Moreover, by using the same siRNA-technique as previous authors [15,22] we achieved the same degree of HOTAIR knockdown, but no induction of *HOXD10* expression in UC cell lines. Most likely, this difference may be due to differences in the endogenous expression patterns and/or methylation patterns across HOX genes between tissues.

A limitation of siRNA-based techniques is that they appear to mostly target the cytoplasmic fraction of lncRNAs, and therefore interfere only partially with their functions in the nucleus (see Additional file 1: Figure S1). Regardless, siRNA- knockdown was used not only in the pioneer studies on HOTAIR [15], but is still state of the art. As an alternative, we therefore used an antisense oligo-nucleotide, which is known to target lincRNAs in the nucleus, to confirm that HOTAIR downregulation did not result in altered *HOXD10* expression. Considering the data from ectopic overexpression and the report of a lack of *HOXD10* induction after HOTAIR knockdown in ovarian cancer cells [23], our finding strongly argues that HOTAIR target genes are tissue-specific.

In addition, most changes in target gene expression occur rather in long-term experiments [15]. Accordingly, stably transfected VM-CUB1 HOTAIR clones showed a significantly increased expression of *HOXD8* and *HOXD9*. However, 5637 clones stably overexpressing HOTAIR were the only instance of a decrease in *HOXD10*. Clearly, HOTAIR target genes in the HOXC and HOXD clusters differ between urothelial and breast carcinoma. As the factors responsible for tissue-specific expression of HOX genes are still poorly understood it will have to be investigated in the future, which factors cause these cell type-dependent effects of HOTAIR on HOX genes.

The effects of HOTAIR overexpression on the phenotype of VM-CUB1 UC cells resembled those by overexpression of HOTAIR in breast cancer [15], gastric cancer [24] and pancreatic cancer [18] cells. They encompassed increased proliferation, anchorage-independent growth and a more mesenchymal morphology with increased migration and invasion capacity. These changes are reflected in the microarray and qRT-PCR gene expression data from VM-CUB1 clones, e.g. increases in cell cycle activating genes. Induction of EMT in VM-CUB1 cells by HOTAIR overexpression was confirmed by E-Cadherin and Vimentin immunofluorescence staining and was reflected in the microarray data by differential expression of genes involved in cell adhesion, cell-matrix-interaction and locomotion, the EMT regulator ZEB1 and downregulation of ΔNp63, a known marker of the epithelial phenotype in UC cell lines [25]. A connection between HOTAIR and EMT was also noted by others [26]. Similarly, modulation of HOTAIR expression by ectopic expression or depletion resulted in changes in migration and invasion capacity in esophageal cancer [17,21], lung cancer cells [16,27] and one bladder cancer cell line (T-24) [14]. Notably, although the phenotypical changes elicited by HOTAIR in VM-CUB1 were similar to those reported in other cancer cells [15,24], they took place in the absence of changes in *HOXD10* expression, demonstrating that HOXD10 is not a general essential mediator of these effects.

Unexpectedly, opposite effects of HOTAIR overexpression were seen in 5637 clones, with decreased proliferation, clonogenicity and migration ability. Across a larger number of UC cell lines, HOTAIR effects varied, with VM-CUB1 and 5637 representing the extremes of the range. Accordingly, we discovered only a relatively small overlap between genes affected by HOTAIR overexpression between these two cell lines and half of these genes changed in opposite directions. Unexpectedly, in the GO analysis, the main biological process positively affected by HOTAIR in 5637 cells was immune response. Most of the associated genes can be upregulated as part of a senescent-associated secretory phenotype (SASP) [28], including several IGFBPs, VEGFA, 11 interleukins, eight CXCL chemokines as well as MMPs and laminins. SASP is induced in senescent cells and accordingly, further markers of senescence, especially the cell cycle inhibitors p21^CIP1^ and p15^INK4B^, were upregulated in HOTAIR-transfected 5637 cells. Due to mutational inactivation of both pRB1 and p53, 5637 cells are unable to undergo regular senescence, but a partial senescence induction accompanied by SASP could account for the observed slower proliferation of HOTAIR-overexpressing 5637 cells. This effect has not yet been reported explicitly in the literature. However, detailed inspection of the effects of HOTAIR on gene expression in pancreatic cancer cells [18], too, reveals several genes involved in immune response, especially interleukins, to be differentially expressed (Figure 6 in Ref. 18). Moreover, in that study, the effects of HOTAIR on the aggressive phenotype of different cell lines were also notably variable, leading the authors to conclude that HOTAIR effects are cell-line dependent [18]. Our findings extend this concept by showing that HOTAIR effects can even turn to the opposite in some cell lines.

The induction of aggressiveness associated with EMT in VM-CUB1 cells was accompanied by losses of the cytokeratins 14 and 5, which have recently been reported to characterize differentiation states in UC [4]. In addition, ΔNp63, a likely regulator of stemness in genitourinary epithelia [29,30], was differentially expressed indicating that aberrant HOTAIR expression may be involved in the establishment of aberrant differentiation states in bladder cancer. Accordingly, genes involved in pattern specification and morphogenesis were differentially expressed in HOTAIR-overexpressing VM-CUB1 cells, including a large number of HOX genes. These effects are obviously highly tissue-dependent, as in other studies fewer differentially regulated HOX genes were reported. Thorough inspection of ChIP data published by Gupta et al. [15] on HOTAIR transfected MDA-MB-231 cells revealed only four HOX genes (*HOXA4*, *C8*, *D10*, *D13*) with increased PRC2 occupancy, although none appeared to gain the repressive H3K27me chromatin mark. In NSCLC cells only *HOXA5* was regulated by HOTAIR [16]. These findings raise the questions how HOTAIR interacts with tissue-specific factors involved in the regulation of HOX patterning and how such differences might be related to the frequent aberrant methylation of various HOX genes in specific cancers [31,32].

Previous publications have emphasized that HOTAIR mediates gene repression by interacting with enzymes catalyzing repressive histone modifications like EZH2 (H3K27 methylation [12,15]) and LSD1 (H3K4 demethylation [33,34]). Thus, many HOTAIR effects depend on EZH2, which is strongly upregulated both in breast and bladder cancers [35]. However, *induction* of gene expression was observed as a consequence of ectopic HOTAIR overexpression in MDA-MB-231 breast cancer cells [15] and in our study at least as many genes became induced as down-regulated in the UC cell lines. While many genes may, of course, become induced indirectly, these findings raise the question of whether and how HOTAIR might mediate gene activation as well. One possible mechanism is relocation of repressive PRC2 or REST/CoREST complexes away from repressed genes, resulting in their reactivation. However, as new interaction partners of HOTAIR continue to emerge [36], it remains possible that the lncRNA can also interact with factors that activate transcription.

Our ChIP analysis for differentially expressed HOX genes revealed that HOTAIR-elicited increases in *HOXC6* and *HOXB2* expression were associated with demethylation of H3K9 and H3K27, respectively. HOTAIR is known to interact with the histone demethylase LSD1, a well known H3K4 demethylase that can also demethylate H3K9 under certain circumstances [33]. The mechanism of H3K27 demethylation remains open, as HOTAIR has not been shown to interact with H3K27 demethylases like UTX. *HOXA11* repression by HOTAIR could be explained by recruitment of LSD1 leading to H3K4 demethylation and *HOXD10* appeared to be regulated via slight increases in H3K27 and H3K9 methylation in accordance with results from the literature [12,15]. Therefore, our data add to the accumulating evidence that HOTAIR might interact with more chromatin modifiers than currently identified.

The most surprising finding of our study is the degree to which HOTAIR function is context-dependent. Obviously, HOTAIR may not be only expressed in a tissue-specific manner, but may also exert tissue-specific effects. Accordingly, comparing our expression profiling results with publicly available data sets for ectopic HOTAIR transfection in cell lines from breast [15], pancreatic [18] and esophageal cancer [21] revealed a minimal overlap between the differentially expressed genes. Even selected candidates shown to mediate HOTAIR-induced phenotypical changes in other studies, e. g. *PCDHB5* or *LAMC2* [15], were differentially expressed among the various compared cell lines. Notably, tissue-specific functions and regulation have also been reported for another cancer-associated lncRNA, MALAT1 [37].

## Conclusions

We report that HOTAIR is overexpressed in many urothelial carcinomas and the highest range of expression is associated with worse clinical parameters. Although these observations concur with those in other cancer types, we have observed that HOTAIR target genes cannot be generalized from one tissue to another and may even differ between individual cancers of the same type. Accordingly, downstream effects of HOTAIR overexpression on cellular identity, phenotype and aggressiveness appear to be more tissue specific than reflected by current literature to date. This conclusion should be considered especially in attempts to use HOTAIR as a therapeutic target.

## Methods

### Patients and tissues

Tissue Set 1 consisted of urothelial cancer and benign tissue samples previously described elsewhere [38], comprising 10 benign bladder tissues and 19 bladder cancer tissues from patients aged from 46 to 86 years (median age: 68 years). Tumor stages and grading according to the current UICC classification were as follows: 3 cases pTa G2, 2 cases pT2 G3, 2 cases pT3 G2, 9 cases pT3 G3 and 3 cases pT4 G3. Tissue samples were collected with patient informed consent and approval by the ethics committee of the medical faculty of the Heinrich Heine University, Study Number 3836. Tissue Set 2 was collected with patient informed consent and approval by the ethics committee of the medical faculty of the University Duisburg-Essen, Study Number 07–3537. This set consisted of 108 cancerous tissues and 7 normal tissues. For clinicopathological parameters see Table 1.

### Cell culture and transfection experiments

Cell lines were provided by Dr. M. A. Knowles (Leeds, UK), Dr. J. Fogh (New York, USA), Dr. B. Grossman (Houston, USA) or by the DSMZ (Braunschweig, Germany). All cell lines were recently verified by DNA fingerprint analysis. Primary urothelial cells (UEC) were prepared from ureters after nephrectomy and were routinely maintained as described [39].

For siRNA-mediated knockdown cells were transfected with 10 nM HOTAIR siRNA (siRNA1 5′-GAACGGGAGUACAGAGAG-3′, siRNA2 5′-UUUUCUACCAGGUGGGUAC-3′) [14,22] or a non-specific control (5′-AGGUAGUGUAAUCGCCUUG-3′) using Lipofectamine RNAiMAX (Invitrogen), according to the manufacturer’s recommendations. Alternatively, we transfected LNA-GapmeRs (EXIQON) for HOTAIR (5′- GCTTCTAAATCCGTT-3′) and a negative control (5′- AACACGTCTATACGC-3′).

For ectopic HOTAIR expression cells were transfected with pLZRS-HOTAIR (Addgene plasmid 26100: LZRS-HOTAIR) [14] or empty vector (Addgene plasmid 31601: LZRS-Rfa) [40] using X-tremeGENE 9 DNA Transfection Reagent (Roche). For generation of stably transfected clones, cells were selected with puromycin.

### Assays for cell viability, clonogenicity and anchorage independent growth

Cell proliferation was measured by means of MTT assay in three independent assays. For clonogenicity assays cells were plated at low density into 10 cm dishes and grown until colonies became visible. Colonies were stained with Giemsa. Anchorage independent growth was investigated by soft-agar assays in 0.4% Noble Agar in 6-well plates in triplicates over three weeks. Colonies were documented by microscopy (Nikon eclipse TE 2000-S).

### Transwell migration and invasion assays

Cells were serum starved (0.5% FCS) overnight and seeded into ThinCerts™ (TC), pore size 8 μm (Greiner Bio-one), in 24-well plates, at 5 × 10^4^ per TC in Opti-MEM (Gibco); bottom chambers were filled with DMEM, 20% FCS. For migration assays TCs were coated with collagen, for invasion assays with matrigel. Three independent assays were performed. Migration capacity was determined after 12 hours, invasion capacity after 24 h for VM-CUB1 cells and 30 h for 5637 cells. Following H&E staining, results were documented with a Nikon Eclipse 400 microscope.

### RNA expression analysis

Total mRNA was isolated using the RNeasy Mini Kit (Qiagen) according to the manufacturer and reverse transcribed using the QuantiTect Reverse Transcription Kit (Qiagen). Also fractionated RNA extraction was performed using the RNeasy Mini Kit (Qiagen) according to a specialized protocol provided by the manufacturer. In brief, cell membranes were lysed by incubation in cold RLN buffer (50 mM Tris pH 8, 140 mM NaCl, 1.5 mM MgCl2, 0.5% Nonidet P40, 1000 U/ml RNAse Inhibitor, 1 mM DTT). After centrifugation (4°C, 300 × g, 2 min) the supernatant containing the cytoplasmic fraction was mixed with RLT puffer (provided with the kit) and 96% Ethanol and applied to the extraction column. The nuclear pellet was lysed in RLT buffer, homogenized using Qiagen shredder columns and applied to the extraction column after mixing with Ethanol. Both fractions were eluted from the column according to the standard protocol of the kit. QRT-PCR was performed using QuantiTect SYBR Green RT-PCR Kit (Qiagen) with self-designed primers (Additional file 2: Table S4) and the housekeeping gene *TBP* (TATA-box binding protein) as a reference. Reactions were carried out on the ABI PRISM® 7900 HT (Life Technologies) instrument.

### Microarray analysis

For microarray analysis we used three independent high quality RNA preparations from VM-CUB1_LV, VM-CUB1_HOTAIR 20, 5637_LV and 5637_HOTAIR 4. Synthesis of cDNA and biotin-labeling of cRNA was performed according to the manufacturers’ protocol (3′ IVT Express Kit; Affymetrix). After fragmentation labelled aRNA was hybridized to Affymetrix PrimeViewTM Human Gene Expression Microarrays for 16 h at 45°C, stained by strepatavidin/phycoerythrin and scanned as described in the manufacturers’ protocol.

Data analyses on Affymetrix CEL files were conducted with GeneSpring GX software (Vers. 12.5; Agilent Technologies; see GEO accession number GSE57672 for data and further description) [41]. Input data pre-processing was concluded by baseline transformation to the median of all samples.

After grouping of samples according to their respective experimental condition (5637, HOTAIR vs. empty vector; VM-CUB1, HOTAIR vs. empty vector; three replicates each) a given probeset had to be expressed above background in all three replicates in at least one of two conditions to be further analysed in pairwise comparisons. Cell line-specific differential gene expression was statistically determined by unpaired *t*-test. Resulting p-values were corrected for multiple testing (Benjamini-Hochberg FDR). The significance threshold was set to p (corr) = 0.01.

Affymetrix PrimeView CEL files, published under GEO accession GSE47638 [21], were processed using GeneSpring GX as described above including probesets expressed above background in at least one of two conditions to be further analysed in pairwise comparison. A fold change cut off of > two-fold was chosen to define differential expression (GSM1153921 vs. GSM1153920, i.e. KYSE180 cells transfected with HOTAIR vector vs. KYSE180 control cells). For comparison with our own expression data we corrected analogous to the procedure used in ref. 21 (p < 0.01 and at least two-fold change).

### Immunofluorescence Stainings

Subsequent to fixation with 4% formaldehyde, cells were permeabilized with 0.1% saponin for 30 minutes (Vimentin) or with ice-cold methanol for 10 min (E-Cadherin). After blocking, primary antibodies were applied to stain Vimentin at 1:400 dilution (#ab92547, abcam) and E-Cadherin at 1:100 dilution (#24E10, Cell Signaling) and incubated for 1 h each. Secondary antibody was diluted 1:500 (Alexa Fluor 488 anti-mouse or -rabbit, Invitrogen). Nuclei were stained by DAPI (1 μg/ml) for 5 minutes. Images were taken on Nikon Eclipse 400 microscope.

### Chomatin Immunoprecipitation

Chomatin immunoprecipitation was performed using the ChIP-IT™ Express Kit (#53008, Active Motif) according to manufacturer’s instructions, except that cells were trypsinized and fixed in 15 ml Greiner tubes. The antibodies used for immunoprecipitation were H3K4me3 (#39915, Active Motif), H3K27me3 (#39535, Active Motif) and H3K9me3 (ab-8898, abcam). Isotype controls were used for background controls. For each immunoprecipitation 20 μg chromatin was used. Sequence content was determined by QPCR (Additional file 2: Table S4).

### Western blotting

Total protein was extracted by lysing the cells in a buffer containing 150 mM NaCl, 1% Triton X-100, 0.5% desoxycholate, 1% Nonidet P-40, 0.1% SDS, 1 mM EDTA, 50 mM Tris (pH 7.6), and protease inhibitor cocktail (10 μL/mL, P-8340, Sigma-Aldrich) for 30 min on ice. Protein concentration was determined by BCA protein assay (Pierce) and samples were separated in SDS-page gels and transferred to PVDF membranes (Millipore). The membrane was blocked with 5% non-fat milk in TBS-T (150 mM NaCl, 10 mM Tris, pH 7.4, and 0.1% Tween-20), washed and then probed with primary antibodies. Antibodies detected EZH2 (Cell Signaling #3147, 1:3000), SUZ12 (Abcam #ab175187, 1:2000) and α-Tubulin (T-5168, Sigma-Aldrich, 1:50 000). After washing, the membrane was incubated with the suitable horseradish peroxidase-conjugated secondary antibody (Santa Cruz, 1:5000) for 1 h and exposed using ECL™ Quantum (Advansta).

## Additional files

Additional file 1: Figure S1. Knockdown efficiency on subcellular fractions of HOTAIR. HOTAIR expression analysis (right) was performed for nuclear and cytoplasmatic fractions from the two cell lines SW-1710 (top) and RT-112 (bottom) subsequent to knockdown of HOTAIR expression by siRNA or LNA-antisense oligos as compared to control samples. Expression of the exclusively nuclear localized lncRNA LIT1 (left) was determined to demonstrate purity of nuclear fractions. Additional file 2:
**Supplementary tables (Table S1-S4).**

## Abbreviations

UC: Urothelial carcinoma
UEC: Uroepithelial cells
LNA ASO: Locked nucleic acid antisense oligo
PRC2: Polycomb repressive complex 2
PcG: Polycomb group proteins
TrX: Trithorax group proteins
H3: Histone 3
K27: Lysine 27
EMT: Epithelial mesenchymal transition
GO: Gene ontology
H&E: hematoxylin & eosin
ChIP: Chromatin immunoprecipitation
SASP: Senescence associated secretory phenotype
NSCLC: Non-small cell lung cancer
FCS: Fetal calf serum
TC: ThinCert TM transwell insert
