# Supplementary material for: The long noncoding RNA HOTAIR has tissue and cell type-dependent effects on HOX gene expression and phenotype of urothelial cancer cells
Source: Mol Cancer. 2015 May 21;14:108. doi: 10.1186/s12943-015-0371-8 (PMC4455698; doi:10.1186/s12943-015-0371-8)
Supplement: Additional file 1: Figure S1. — Knockdown efficiency on subcellular fractions of HOTAIR. HOTAIR expression analysis (right) was performed for nuclear and cytoplasmatic fractions from the two cell lines SW-1710 (top) and RT-112 (bottom) subsequent to knockdown of HOTAIR expression by siRNA or LNA-antisense oligos as compared to control samples. Expression of the exclusively nuclear localized lncRNA LIT1 (left) was determined to demonstrate purity of nuclear fractions. [file 12943_2015_371_MOESM1_ESM.pdf]

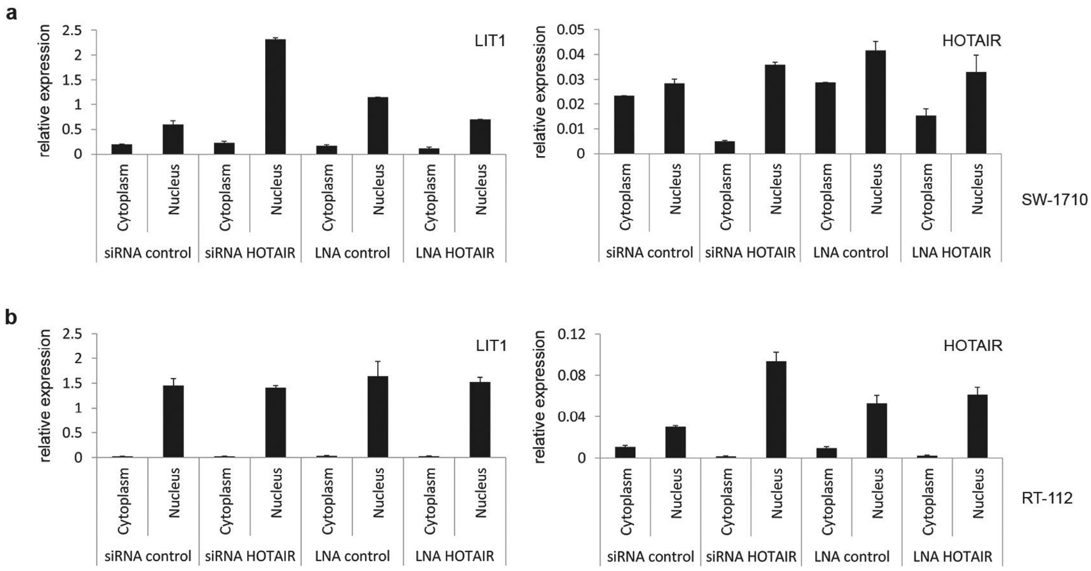

Supplementary Figure 1: Knockdown efficiency on subcellular fractions of HOTAIR

HOTAIR expression analysis (right) was performed for nuclear and cytoplasmatic fractions from the two cell lines SW-1710 (top) and RT-112 (bottom) subsequent to knockdown of HOTAIR expression by siRNA or LNA-antisense oligos as compared to control samples. Expression of the exclusively nuclear localized lncRNA LIT1 (left) was determined to demonstrate purity of nuclear fractions.
