# Supplementary material for: The long noncoding RNA HOTAIR has tissue and cell type-dependent effects on HOX gene expression and phenotype of urothelial cancer cells
Source: Mol Cancer. 2015 May 21;14:108. doi: 10.1186/s12943-015-0371-8 (PMC4455698; doi:10.1186/s12943-015-0371-8)
Supplement: Additional file 2: — Supplementary tables (Table S1-S4). [file 12943_2015_371_MOESM2_ESM.pdf]

Supplementary Table 1: Results from statistical analysis of MTT assays for additional stably transfected HOTAIR clones of VM-CUB1 and 5637. P-values were determined by t-test for every clone compared to vector clone at the given time points.

**Day**	**VM-CUB1 HOTAIR 20**	**VM-CUB1 HOTAIR 3**	**VM-CUB1 HOTAIR 4**	**VM-CUB1 HOTAIR 5**	**VM-CUB1 HOTAIR 9**
2	< 0.05	n.s.	< 0.05	n.s.	n.s.
3	< 0.05	n.s.	n.s.	< 0.001	< 0.05
4	< 0.05	n.s.	n.s.	n.s.	n.s.
**Day**	**5637 HOTAIR 4**	**5637 HOTAIR 16**	**5637 HOTAIR A10**	**5637 HOTAIR A14**	
2	< 0.05	n.s.	n.s.	< 0.05	
3	<0.001	< 0.001	< 0.05	< 0.001	
4	<0.001	< 0.001	n.s.	< 0.05	

n.s.- not significant

Supplementary Table 2: Results of gene ontology group analysis using DAVID for differentially expressed genes with p< 0.01 and at least 2- fold expression changes

**VM-CUB1 HOTAIR vs Vector**	**5637 HOTAIR vs Vector**
**GO downregulated genes**	
regulation of apoptosis	protein-DNA complex
cell-cell adhesion	cellular macromolecular complex subunit organization
macromolecular complex subunit organization	chromatin organization
cell cycle	RNA processing
regulation of specific transcription from RNA polymerase II promoter	regulation of transcription from RNA polymerase II promoter
	regulation of cell cycle
**GO upregulated genes**	
regulation of transcription	response to wounding
regulation of RNA metabolic process	inflammatory response
regulation of locomotion	cytokine activity
Homeobox protein, antennapedia type	chemotaxis
regulation of growth	positive regulation of signal transduction
pattern specification process	regulation of programmed cell death

Supplementary Table 3: Results of validation experiments obtained by quantitative RT-PCR and evidence for dose dependency with intermediate HOTAIR expression ($p < 0.05$)

	**Microarray VM-CUB1**	**qRT-PCR VM-CUB1 HOTAIR**	**Dose dependency**	**Microarray 5637**	**qRT-PCR 5637 HOTAIR**	**Dose dependency**
HOXA1	2.7 x down	down	yes	1.7 x down	down	yes
HOXA11	44.6 x down	down	yes	--	down	yes
HOXA13	2.6 x up	up	no	--	n.c.	no
HOXB2	1.8 x up	up	no	2.8 x up	up	no
HOXB13	3.0 x up	up	no	--	n.c.	no
HOXC6	32.1 x up	up	no	1.4 x down	n.c.	no
HOXD3	6.6 x up	up	yes	1.7 x down	down	no
HOXD9	4.3 x up	up	no	1.4 x down	down	yes
HOXD10	--	n.c.	no	2.0 x down	down	no
KRT14	3.1 x down	down	no	--	up	no
KRT5	182 x down	down	no	1.9 x up	up	no
p63/ΔN-p63	90 x down	down	no	1.6 x up	up	no
E-Cadherin/ CDH1	84.1 x down	down	no	2.2 x up	up	no
Vimentin/ VIM1	1243 x up	up	yes	--	down	yes
ZEB1	39 x up	up	no	--	n.c.	no
PCDHB5	8.2 x down	down	no	--	n.c.	no
LAMB3	4.8 x down	down	yes	--	n.c.	no
p57KIP2/CDKN1C	--	up	no	17 x up	up	yes
p14/CDKN2A	3.1 x down	down	yes	--	n.v.	no
p15/CDKN2B	2.2 x down	down	yes	1.6 x up	up	no
p16/ CDKN2A	3.1 x down	down	no	--	n.c.	no
p21/ CDKN1A	--	n.c.	no	2.3 x up	up	yes

n.c.: no change, -- not detected

Supplementary Table 4: Purpose, targets, and sequences of used oligonucleotides. Forward and reverse primer sequences are given in 5´ to 3´orientation.

Purpose	Target	Sequence
qRT-PCR	HOTAIR	GGTAGAAAAAGCAACCACGAAGC
		ACATAAACCTCTGTCTGTGAGTGCC
	HOXA1	GCTCACAGAACTTCAGTGCGC
		CAGTTCCGTGAGCTGCTTGG
	HOXA11	CAGCAGAGGAGAAAGAGCGG
		TCGGATCTGGTACTTGGTATAGG
	HOXA13	CTGGAACGGCCAAATGTACTG
		CTTGGTATAAGGCACGCGCTT
	HOXB2	CGAGTTCCCTTGGATGAAAGAG
		CGGCAGTCCCAGGCCAT
	HOXB13	CAGCCAGATGTGTTGCCAGG
		CAACTGCCCCTTGCTGTTACG
	HOXC6	CAGGACCAGAAAGCCAGTATC
		TCTGGTACCGCGAGTAGATC
	HOXC11	CCCGGATGCTGAACCTGA
		AGCCGGTCTCTGCTCAGT
	HOXC12	CCGGCAGCTTGGTATCGC
		CGAGAGCGGCTGTTGATCG
	HOXC13	TCAGCGGGCACCCGGAG
		CCGGTAGCTGCTCACCTCG
	HOXD3	CAATGCTTCTAGCTCCTCAGCC
		TCTTGTCCTCGCAGCTCTCTC
	HOXD9	CCCAGGCTGTTCGCTGAAG
		GCGTGGATCCAGTTCGCG
	HOXD10	GAGCTGCCTGGCTGAGG
		GCAGTGAGCCAATTGCTGG
	HOXD11	CAGAAGGCAGCGGTGGCG
		GTACTTGGTATAGGGACAGCGC
	HOXD12	AGCAGATTGCGGAGTTGG
		CTTGCTGGTCGCTGAGG
	HOXD13	GACATGGTGTCCACTTTCGG
		GTAGACGCACATGTCCGGC
	E-Cadherin/ CDH1	ACCAGAATAAAGACCAAGTGACCA
		AGCAAGAGCAGCAGAATCAGAAT
	VIMENTIN/ VIM1	AATGGCTCGTCACCTTCGTGAAT
		CAGATTAGTTTCCCTCAGGTTCAG
	ZEB1	TCACATCAAATCAGGAAGAGATC
		CACTGTGAATTCTTAAGTGCTC
	PCDHB5	AGGTGTGTTTGACCGGAGAC
		TCCCTATTTCTTCACCAGCGC
	LAMB3	CAAGCCTGAGACCTACTGC
		GCCACATTCTCTACTCGGTG

	KRT5	GATGATCCAGAGGCTGAGAGC
		CTCGGCCAGCTTGTTCCTG
	KRT14	GCGCACCATGCAGAACCTG
		CCTCCACGCTGCCAATCATC
	p21/ CDKN1A	GGAAGACCATGTGGACCTGT
		GGCGTTTGGAGTGGTAGAAA
	p57/ CDKN1C	GCGGCGATCAAGAAGCTG
		CGACGACTTCTCAGGCGC
	p14/ CDKN2A	GTGGCCCTCGTGCTGATG
		CAGCAGCTCCGCCACTC
	p16/ CDKN2A	CAACGCACCGAATAGTAACG
		AGCACCACCAGCGTGTC
	p15/ CDKN2B	GCGGGACTAGTGGAGAAGG
		GCCTCCCGAACGGTTGA
	p63	AAACCCAGCTCATTTCTCTT
		CAATGGGCTGAACTGAACATA
	ΔN p63	CGCCATGCCTGTCTACAAAA
		TCTTCCTGTGATGGGATCTTC
	LIT1	CCC TGC TGT GCC TTC AGC CC
		CCA GGC TGC CTC ACC CAA CG
	EZH2	AAT GTG TCC TGC AAG AAC TG
		CAT TTT TCT GCA CAG GAT CTT T
	SUZ12	AGCAAGAGATGACCTGCATTGC
		CAGGTTGGCGATGAATATCCTG
ChIP	HOXD10	GGGCACTCTGGCTTCCCT
		CAGCTCTAGGGGCCTCCA
	HOXC6	CGATTGTGTTGTCCTGATGTC
		CCAGTTCCATTAGCCAAGAGA
	HOXA11	GGGGTGGTGGTAGACGTT
		GAGGAGCTCGTGCACAGA
	HOXB2	GGTCGCAGATGTCCTCCA
		GAAGCACAAGCGGCAGAC
	GAPDH	TACTAGCGGTTTTACGGGCG
		TCGAACAGGAGGAGCAGAGAGCGA
	TBP	GGAGTGGCAGAACTGAAT
		GCAAGAACGCTAGCTGAT
	GRM6	GAGAGGGACGCTGGACAC
		CTCCGTCTCCATCATGGTC
